# Supplementary material for: Multicore fiber optic imaging reveals that astrocyte calcium activity in the cerebral cortex is modulated by internal motivational state
Source: bioRxiv. 2023 May 18:2023.05.18.541390. Preprint. [Version 1] doi: 10.1101/2023.05.18.541390 (PMC10245653; doi:10.1101/2023.05.18.541390)
Supplement: Supplement 1 [file NIHPP2023.05.18.541390v1-supplement-1.pdf]

bioRxiv preprint doi: https://doi.org/10.1101/2023.05.18.541390; this version posted May 18, 2023. The copyright holder for this preprint (which was not certified by peer review) is the author/funder, who has granted bioRxiv a license to display the preprint in perpetuity. It is made available under aCC-BY 4.0 International license.

Supplemental Items

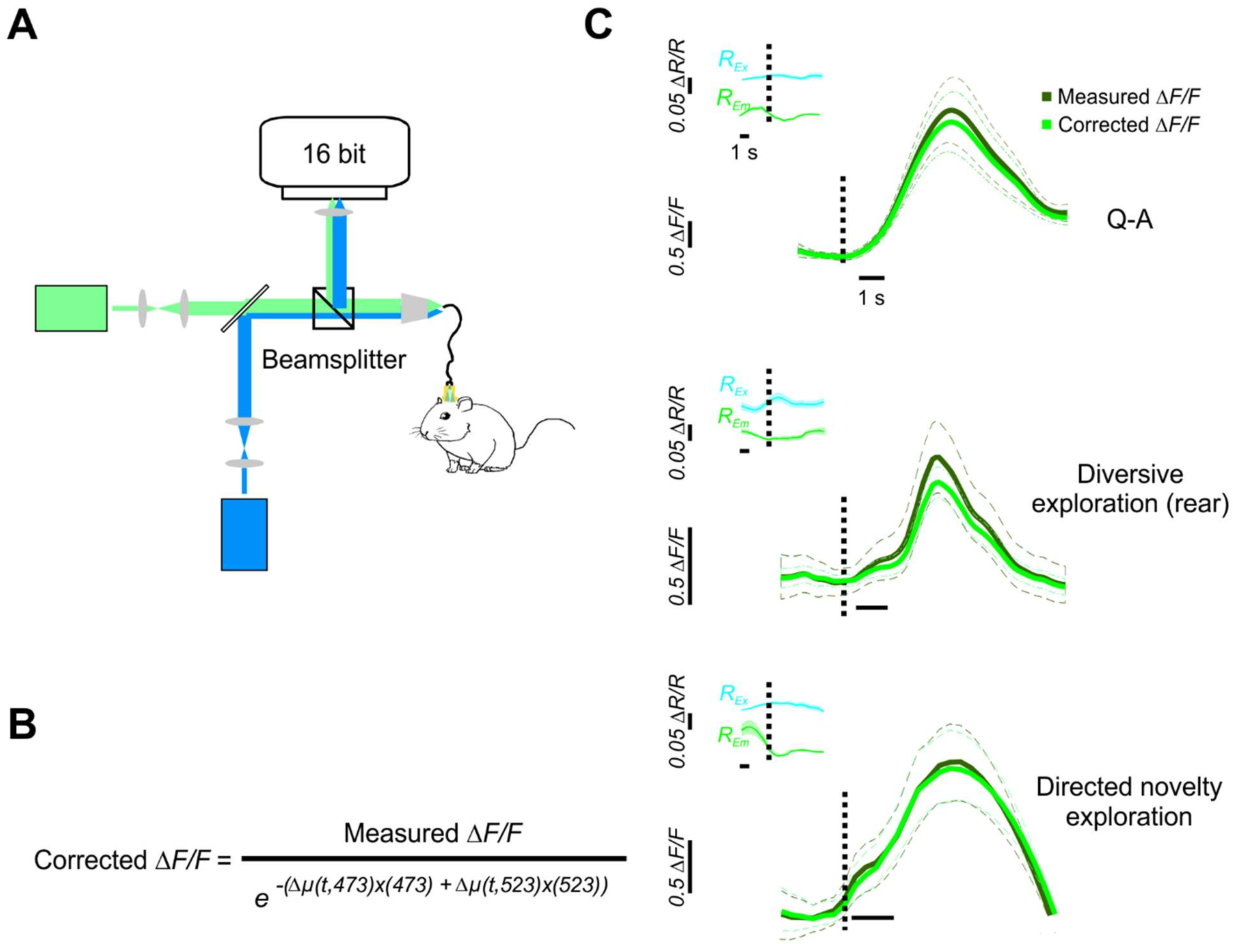

bioRxiv preprint doi: https://doi.org/10.1101/2023.05.18.541390; this version posted May 18, 2023. The copyright holder for this preprint (which was not certified by peer review) is the author/funder, who has granted bioRxiv a license to display the preprint in perpetuity. It is made available under aCC-BY 4.0 International license.

Figure S1. Imaging reflectance change during behaviors.

(A) Freely moving reflectance imaging set up at 473 nm and 523 nm, peak excitation/emission wavelengths used for GCaMP imaging. (B) Formula for correcting GCaMP fluorescence signals for associated hemodynamic changes. (C) Reflectance changes had a small effect on the amplitude and time course of GCaMP fluorescence signals in the major behaviors studied in this paper.
